# Migration and transformation of coastal wetlands in response to rising seas

**DOI:** 10.1126/sciadv.abo5174

**Published:** 2022-06-29

**Authors:** Michael J. Osland, Bogdan Chivoiu, Nicholas M. Enwright, Karen M. Thorne, Glenn R. Guntenspergen, James B. Grace, Leah L. Dale, William Brooks, Nate Herold, John W. Day, Fred H. Sklar, Christopher M. Swarzenzki

**Affiliations:** 1 U.S. Geological Survey, Wetland and Aquatic Research Center, Lafayette, LA, USA.; 2 Cherokee Nation System Solutions, contracted to the U.S. Geological Survey, Wetland and Aquatic Research Center, Lafayette, LA, USA.; 3 U.S. Geological Survey, Western Ecological Research Center, Davis, CA, USA.; 4 U.S. Geological Survey, Eastern Ecological Science Center, Laurel, MD, USA.; 5 National Oceanic and Atmospheric Administration, Office for Coastal Management, Charleston, SC, USA.; 6 Louisiana State University, Baton Rouge, LA, USA.; 7 South Florida Water Management District, West Palm Beach, FL, USA.; 8 U.S. Geological Survey, Lower Mississippi-Gulf Water Science Center, Baton Rouge, LA, USA.

## Abstract

Coastal wetlands are not only among the world’s most valued ecosystems but also among the most threatened by high greenhouse gas emissions that lead to accelerated sea level rise. There is intense debate regarding the extent to which landward migration of wetlands might compensate for seaward wetland losses. By integrating data from 166 estuaries across the conterminous United States, we show that landward migration of coastal wetlands will transform coastlines but not counter seaward losses. Two-thirds of potential migration is expected to occur at the expense of coastal freshwater wetlands, while the remaining one-third is expected to occur at the expense of valuable uplands, including croplands, forests, pastures, and grasslands. Our analyses underscore the need to better prepare for coastal transformations and net wetland loss due to rising seas.

## Teaser

Landward migration of coastal wetlands under rising seas will transform coastlines but not compensate for seaward losses.

## INTRODUCTION

Societal perspectives of the value of coastal wetlands have changed greatly in the past century (*1*). Coastal wetlands that were once seen as flooded wastelands needing to be drained or filled are now considered among the most valuable ecosystems on the planet due to their support of biodiversity and critical ecosystem services (*2*). In addition to providing fish and wildlife habitat, coastal wetlands protect coastal communities from storms, slow erosion, sequester carbon, support productive fisheries, improve water quality, and provide recreational opportunities (*3*). Despite their tremendous ecological and societal value, coastal wetlands are now threatened by accelerated sea level rise (*4*, *5*) largely driven by high greenhouse gas emissions (*6*, *7*).

Some coastal wetlands can adapt to rising seas via landward migration, where coastal wetlands move landward into adjacent upslope or upriver ecosystems in response to changing inundation and salinity regimes (*8*–*10*). Of central concern, however, is the extent to which landward migration could compensate for seaward wetland losses and mitigate for the loss of biodiversity and ecosystem services. Several recent analyses have indicated that unimpeded landward migration may actually lead to large wetland gains despite seaward wetland losses (*8*, *9*, *11*), but this potential for wetland gains via landward migration has been questioned, in part, by the inability to fill increased accommodation space (*12*). Resolving this disagreement has also been hindered by the limited scope of past efforts (*8*, *9*, *11*), which, due to data constraints, have typically focused solely on the most seaward coastal wetlands (i.e., tidal saline wetlands) without explicitly quantifying the transformative impacts to adjacent lands, including coastal freshwater wetlands and valuable coastal uplands. In particular, by excluding coastal freshwater wetlands, these studies have underestimated the potential for net loss of wetlands. Here, we investigate the following four questions across the conterminous United States: (i) Where can wetland loss and migration occur? (ii) Which adjacent upland and freshwater wetland classes are most vulnerable to wetland migration? (iii) Can landward migration compensate for seaward wetland losses? (iv) Where are hot spots for wetland migration into adjacent uplands?

The potential for wetland adaption to sea level rise is governed partly by the availability of accommodation space, which is the vertical and lateral space available for sediment filling, organic matter accumulation, and wetland establishment in response to rising seas (*12*–*14*). There are two primary mechanisms that have enabled coastal wetlands to adapt to past increases in sea level. First, biogeomorphic feedbacks between inundation, plant growth, and sedimentation can build wetland elevation (*15*–*18*). Across the world, there are many examples of wetlands that have relied on this mode of vertical accretion to adjust to moderate rates of sea level rise. However, there are limits to the potential for wetland elevation gains via vertical accretion. For example, recent syntheses have indicated that catastrophic coastal wetland drowning is likely when decadal-scale, sustained sea level rise rates exceed upper thresholds for vertical accretion, which have been identified as 7, 6, and 6 to 9 mm/year for the U.K. marshes (*19*), global mangroves (*20*), and Louisiana (U.S.) marshes (*21*), respectively. For most regions, these tipping points could be exceeded within the next 50 years under sea level rise scenarios associated with high greenhouse gas emissions (*19*–*21*). In Louisiana’s Mississippi River Delta, these tipping points are being exceeded now, as widespread wetland loss is already occurring (*22*) due to high rates of subsidence and relative sea level rise (*23*).

**Fig. 1.**
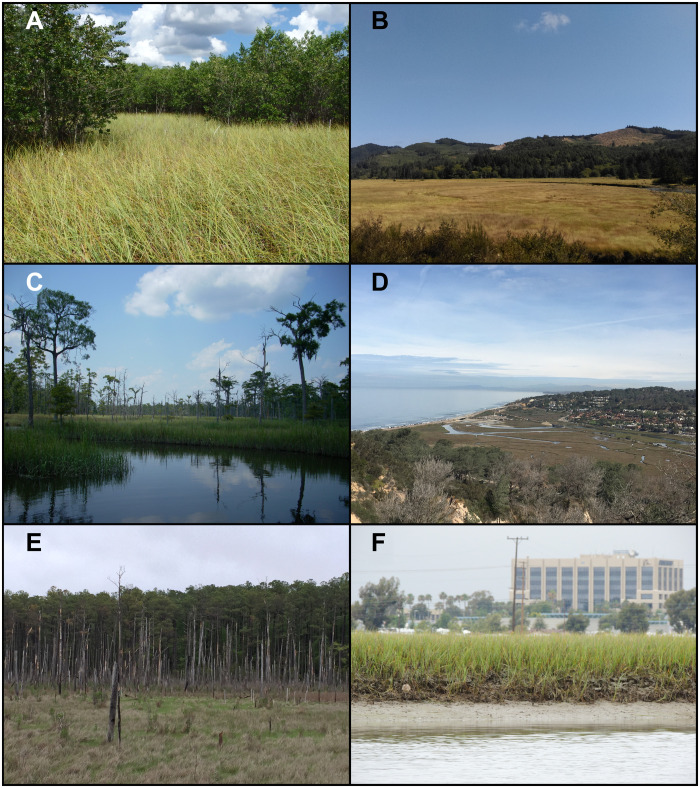
To adapt to rising sea levels, tidal saline wetlands can migrate landward at the expense of adjacent freshwater wetlands and upland ecosystems, but migration can be hindered by natural and anthropogenic barriers. Examples from the conterminous United States include the following: (**A**) mangrove forest migration into a freshwater marsh in south Florida, (**B**) marsh migration constrained by topographic barriers in an Oregon estuary, (**C**) marsh migration into a freshwater forested wetland in South Carolina, (**D**) marsh migration constrained by topographic and urban barriers in southern California, (**E**) marsh migration into an upland forest in Maryland, and (**F**) marsh migration constrained by urban barriers in southern California. Photo credits: Michael J. Osland (A), Karen M. Thorne (B, D, and F), Ken W. Krauss (C), and Glenn R. Guntenspergen (E).

Wetland landward migration is a second mechanism for adaptation to sea level rise (*8*–*10*) and the primary focus of this study. The landward movement of wetlands comes at the expense of adjacent lands that also provide ecological and societal benefits. For example, rising sea levels and saltwater intrusion cause wetlands to encroach on and replace valuable uplands, including agricultural croplands, pastures, terrestrial forests, and grasslands (*24*, *25*). Landward migration of saline wetlands also threatens adjacent freshwater wetland forests and marshes (*26*–*28*). Most wetland-rich estuaries contain dynamic mixtures of highly productive saline, brackish, and freshwater wetlands, where ecological transitions (ecotones) are governed by abiotic and biotic factors that vary across land-ocean interfaces (see Fig. 1). For example, in the iconic Everglades wetlands of south Florida (U.S.), saline mangrove forests transition to brackish and freshwater marshes across subtle inundation and salinity gradients (*29*). Similarly, along the northern coast of the Gulf of Mexico in Louisiana’s vast Mississippi River Delta (U.S.), salinity and inundation gradients produce ecological transitions that extend from salt marshes to freshwater wetland forests (*30*). Similar to their saline counterparts, valuable inland tidal freshwater marshes and forests provide many ecosystem services to coastal communities (*31*) but can be transformed into saline wetlands or open water by landward migration of wetlands driven by sea level rise and saltwater intrusion (*26*, *27*).

Here, we examine the potential for the landward migration of coastal wetlands within 166 estuarine drainage areas that span the coastal conterminous United States, along the Pacific Ocean, northern Gulf of Mexico, and Atlantic Ocean using regional relative sea level projections (*32*) developed for the United States based on an Intermediate-High, 1.5-m global mean sea level rise scenario (*5*, *32*). These projections incorporate vertical land movement (for example, subsidence or uplift) and other factors that influence local relative sea level rise rates (*32*). Because of regional differences in geomorphology, climate, and management of coastal lands, there is much variation in the structure, function, and area of coastal wetland transitional zones (Fig. 1). Salt marshes and mangrove forests are tidal saline wetlands that are common in different regions (*33*–*36*). Coastal freshwater wetlands located on the landward side of tidal saline wetlands include tidal and nontidal freshwater wetland forests and marshes (*31*, *37*, *38*). The upland transitional areas adjacent to coastal wetlands include coastal forests, grasslands, agricultural croplands, pastures, and urban lands. While some coastal wetlands are surrounded by topographic barriers that impede migration, others are located along low-lying coasts with gradual transitions that span saline wetlands, brackish wetlands, freshwater wetlands, and adjacent low-lying upland ecosystems. Our landward migration analyses focus on the upslope and upriver movement of two broad wetland classes: (i) tidal saline wetlands and (ii) freshwater wetlands. The freshwater wetland class includes tidal and nontidal freshwater wetlands. Within estuarine drainage areas, we quantified the potential for wetland migration into four upland categories (terrestrial forest, terrestrial grassland, agricultural croplands, and pasture) and two freshwater wetland categories (freshwater forested wetland and freshwater marsh).

## RESULTS AND DISCUSSION

### Where can wetland loss and migration occur?

In figs. S1 and S2, we illustrate the potential for loss of tidal saline wetlands and freshwater wetlands across the conterminous United States under a worst-case scenario, where saltwater intrusion leads to the collapse of freshwater wetlands (*39*–*41*) and biogeomorphic feedbacks are not able to compensate for high rates of sea level rise (*19*–*21*), partially due to the inability to fill emerging accommodation space (*12*). The risk of catastrophic, landscape-scale wetland loss is especially high along the Gulf of Mexico and south Atlantic coasts, with hot spots in the Mississippi River Delta, Everglades, Albemarle-Pamlico, and Chesapeake Bay estuaries. The five states with the highest potential for wetland loss are Louisiana (29%), Florida (25%), North Carolina (10%), Texas (8%), and South Carolina (7%), which collectively account for 79% of the total potential wetland loss (figs. S1 and S2).

**Fig. 2.**
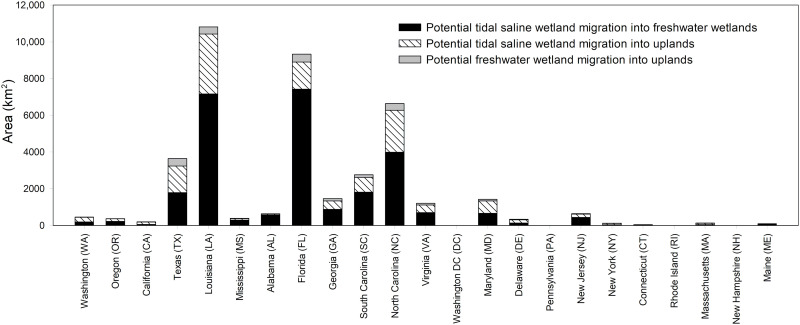
Potential wetland landward migration in response to sea level rise across the conterminous United States. Data are presented for 22 coastal states and Washington, DC in three categories—tidal saline wetland migration into freshwater wetlands, tidal saline wetland migration into uplands, and freshwater wetland migration into uplands.

**Fig. 3.**
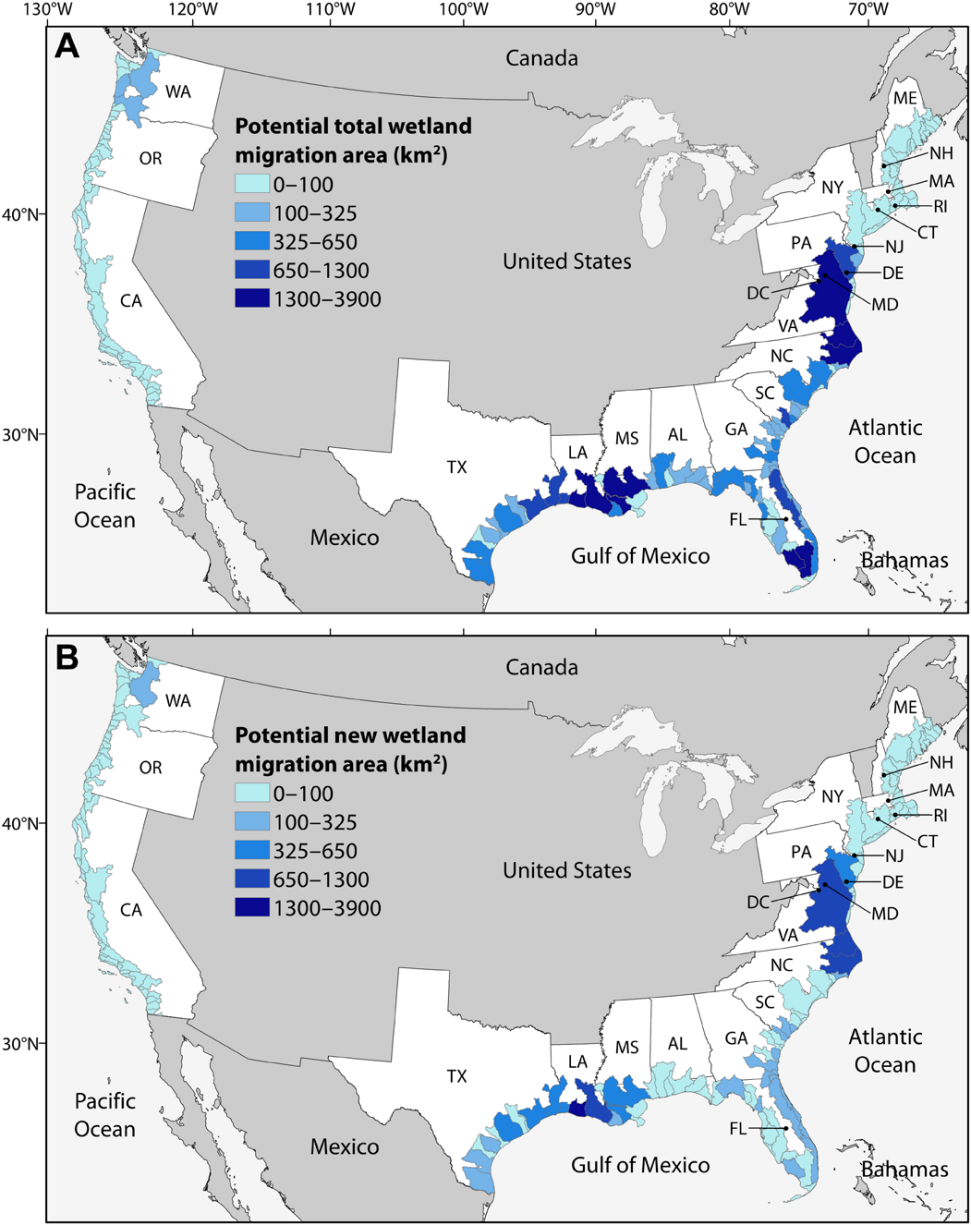
Maps of potential wetland landward migration in response to sea level rise across the conterminous United States, within 166 estuarine drainage areas. (**A**) Map of total potential wetland migration within estuarine drainage areas. (**B**) Map of the potential for new coastal wetland formation due to wetland migration into uplands within estuarine drainage areas. Note that the 166 polygons on the map are estuarine drainage areas and the colors reflect the amount of potential migration within each polygon but not the inland extent of potential migration.

**Fig. 4.**
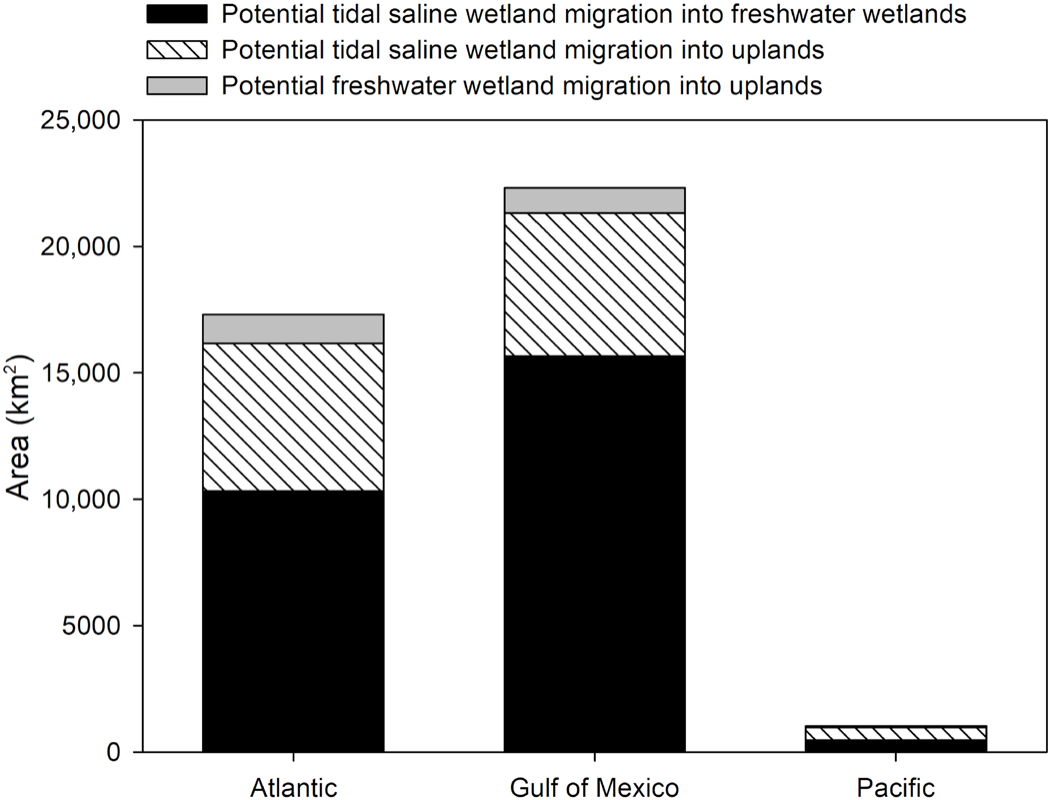
Potential for coastal wetland migration along the Atlantic, Gulf of Mexico, and Pacific coasts of the conterminous United States. Wetland migration potential is presented in three categories: (i) tidal saline wetland migration into freshwater wetlands, (ii) tidal saline wetland migration into uplands, and (iii) freshwater wetland migration into uplands.

Across the conterminous United States, most of the area available for wetland migration is located along the northern Gulf of Mexico and the southern to mid-Atlantic coasts, with much less land available on the Pacific and northern Atlantic (New England) coasts (Figs. 2 to 4 and tables S1 and S2). Our study quantifies and compares the potential for landward migration across all three coasts. Physiography explains the high potential for migration along the northern Gulf of Mexico and the southern to mid-Atlantic coasts, which contain extensive, low-lying coastal plains. In contrast, the steep topographies along the Pacific and northern Atlantic coasts hinder wetland migration. The five states with the most potential for wetland migration are Louisiana (27%), Florida (23%), North Carolina (16%), Texas (9%), and South Carolina (7%), which collectively account for 82% of the total area available for migration (Fig. 2). Of the 30 estuaries with the highest potential for wetland migration, eight are in Florida and seven are in Louisiana (Fig. 3 and table S1).

### Which adjacent upland and freshwater wetland classes are most vulnerable to wetland migration?

**Fig. 5.**
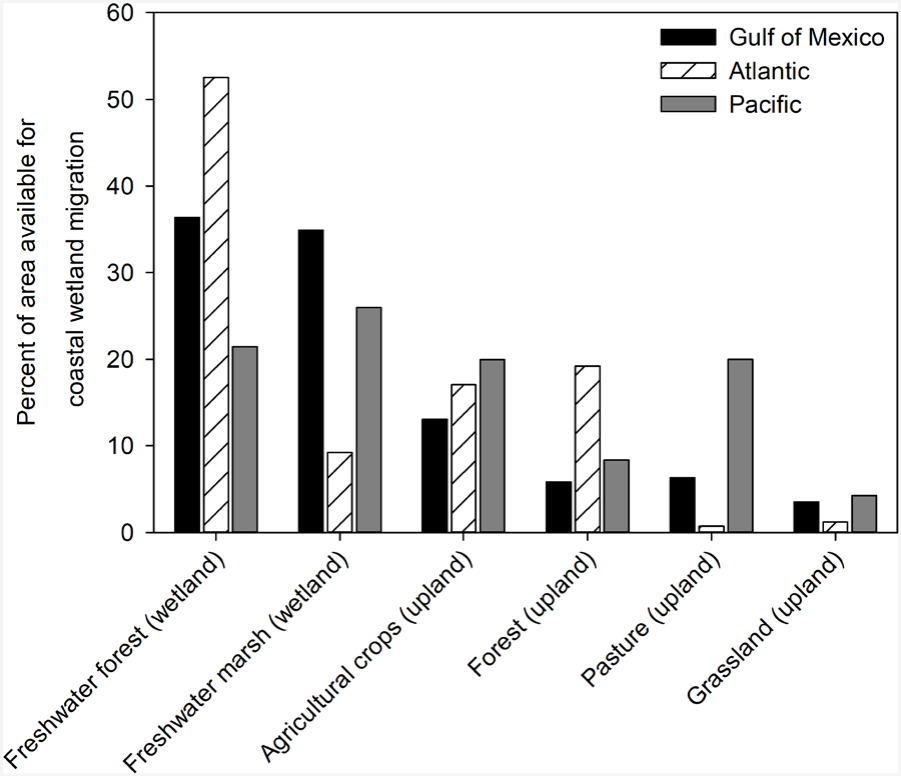
Relative vulnerability of freshwater wetlands and uplands to coastal wetland migration in response to rising sea levels. Data are shown for the Gulf of Mexico, Atlantic, and Pacific coasts of the conterminous United States and divided into two freshwater wetland and four upland classes. The vertical axis reflects the coast-specific percent of the area available for coastal wetland migration.

**Fig. 6.**
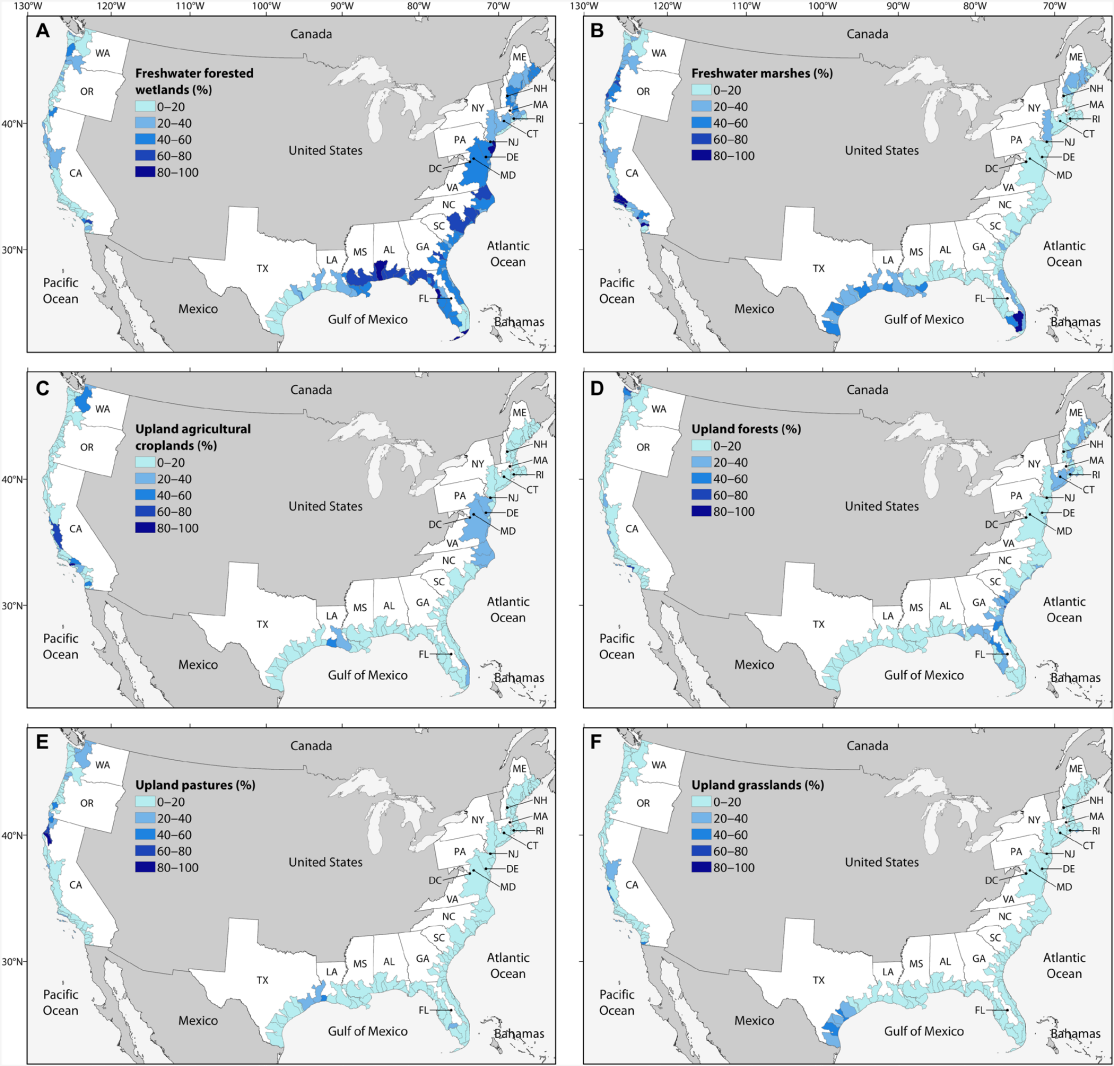
Relative vulnerability of freshwater wetlands and uplands to coastal wetland migration in response to rising sea levels. (**A**) Potential for coastal wetland migration into freshwater forested wetlands. (**B**) Potential for coastal wetland migration into freshwater marshes. (**C**) Potential for coastal wetland migration into upland agricultural croplands. (**D**) Potential for coastal wetland migration into upland forests. (**E**) Potential for coastal wetland migration into upland pastures. (**F**) Potential for coastal wetland migration into upland grasslands. Data are shown for estuarine drainage areas, and the legends reflect the percent of the area available for coastal wetland migration within each estuarine drainage area.

Our analyses indicate that freshwater forested wetlands and freshwater marshes collectively represent two-thirds of the total area available for wetland migration across the conterminous United States (Figs. 5 and 6, A and B). Upland agricultural croplands, forests, pastures, and grasslands collectively represent one-third of the total area available for wetland migration (Figs. 5 and 6, C to F). Across the three coasts, there are differences in the relative vulnerability of upland and wetland classes to landward migration. For example, freshwater forested wetlands, freshwater marshes, and upland croplands are the three most vulnerable classes to conversion along the northern Gulf of Mexico coast (Figs. 5 and 6). Along the Atlantic coast, freshwater forested wetlands, upland forests, and upland croplands are most vulnerable (Figs. 5 and 6). In contrast, the comparatively small amount of land available for wetland migration along the Pacific coast is more evenly distributed among the six classes (Figs. 5 and 6).

### Can landward migration compensate for seaward wetland losses?

**Fig. 7.**
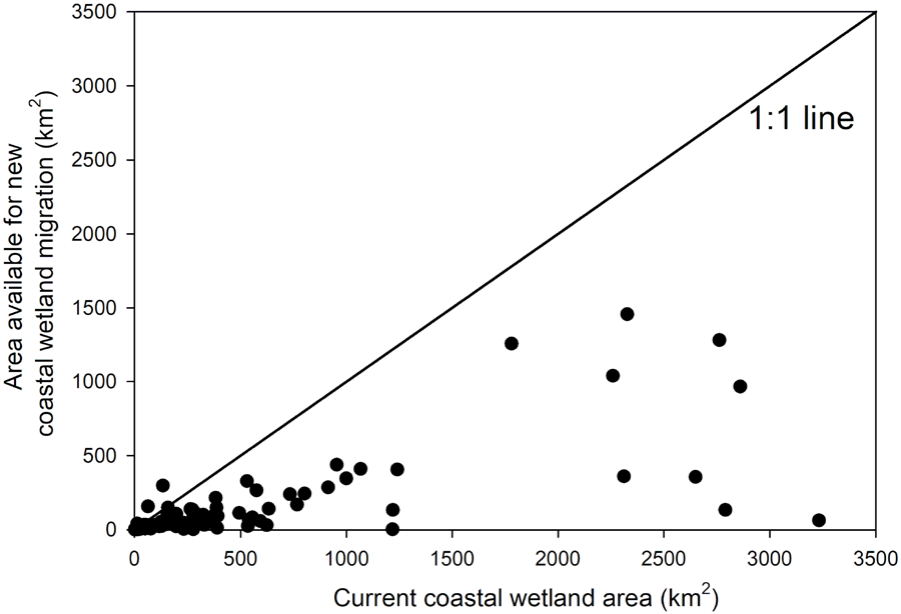
Comparison of current coastal wetland area to the area available for new coastal wetland migration. Each circle represents an individual estuarine drainage area. The 1:1 line denotes where current wetland area is equivalent to the area available for wetland migration. This figure includes all kinds of coastal wetlands together (that is, tidal saline wetlands and freshwater wetlands).

**Fig. 8.**
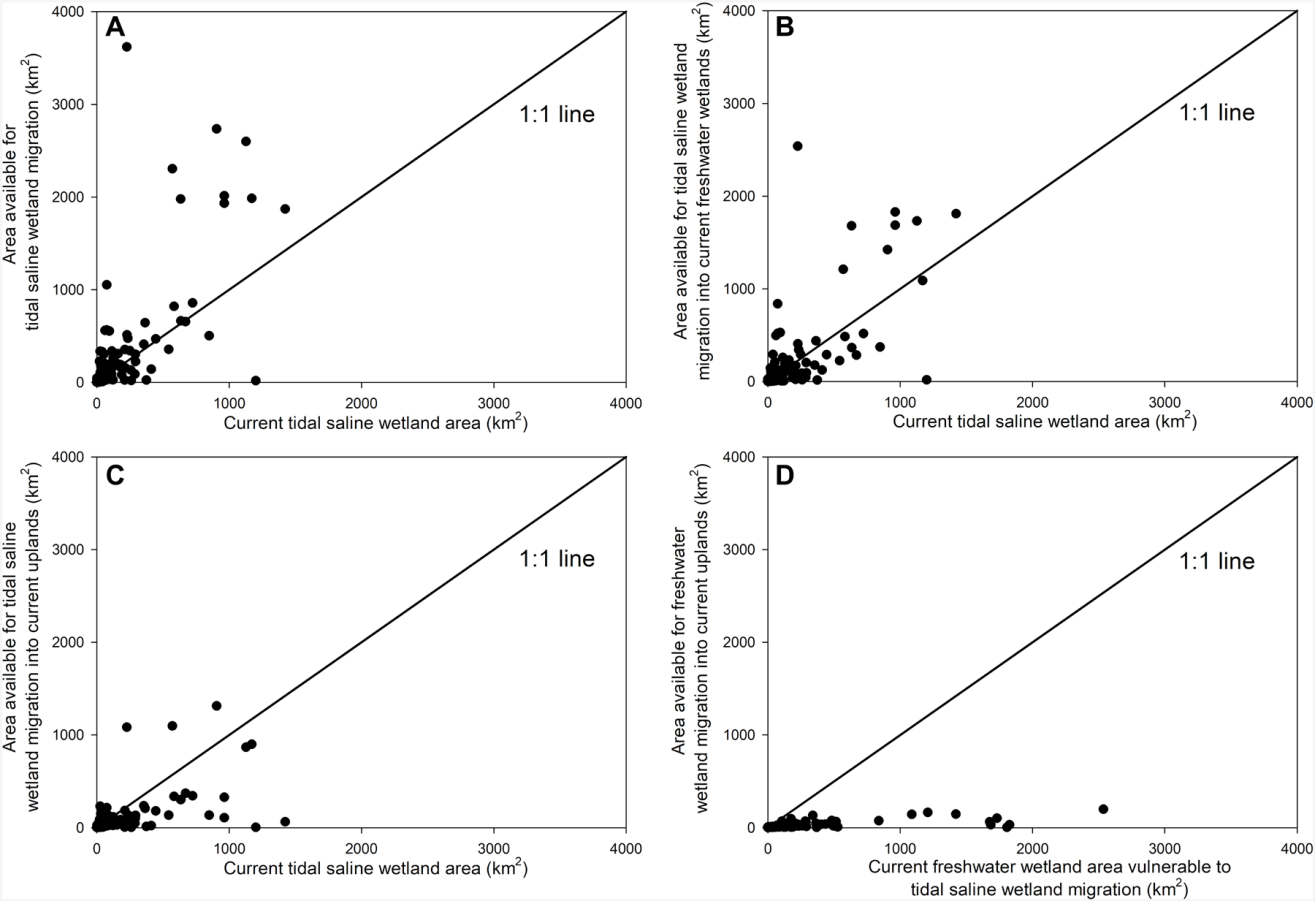
Comparisons of current wetland area to area available for future wetland migration. (**A**) Current tidal saline wetland area compared to the total area available for tidal saline wetland migration. (**B**) Current tidal saline wetland area compared to the area available for tidal saline wetland migration into current freshwater wetlands. (**C**) Current tidal saline wetland area compared to the area available for tidal saline wetland migration into current uplands. (**D**) Current freshwater wetland area compared to the area available for freshwater wetland migration into current uplands. Each circle represents an individual estuarine drainage area. The 1:1 lines denote where current wetland area is equivalent to the area available for wetland migration.

One of the critical limitations of prior landward migration studies is that they have focused exclusively on tidal saline wetlands without explicit consideration of adjacent freshwater wetlands (*8*, *9*, *11*). By considering all coastal wetlands together (that is, saline, brackish, and freshwater wetlands), our analyses show that the area available for landward migration of all coastal wetland classes is considerably less than the current coastal wetland area (Fig. 7). Although the area available for potential migration of tidal saline wetlands is larger than the current tidal saline wetland area (Fig. 8A), most of this migration occurs at the expense of coastal freshwater wetlands (Figs. 6, A and B, and 8B). There is less room for tidal saline wetlands or freshwater wetlands to migrate landward into adjacent uplands (Fig. 8, C and D, respectively). When considering all coastal wetlands together, landward migration cannot compensate for seaward wetland losses (Fig. 7).

Some freshwater wetlands can migrate landward along river corridors into adjacent freshwater wetlands. However, that migration does not result in new wetland formation because it occurs at the expense of other freshwater wetland classes. Coastal freshwater wetlands that cannot migrate landward into uplands due to topographic barriers are particularly vulnerable to rising sea levels that lead to the landward migration of tidal saline wetlands (Fig. 8). For example, in the Mississippi River Delta, Chesapeake Bay, Albemarle-Pamlico, and Columbia River estuaries, salt marshes can migrate landward at the expense of freshwater marshes and freshwater wetland forests, but those same freshwater wetlands cannot form new wetlands via landward migration into uplands due to the presence of topographic barriers (Fig. 8 and table S1). Similarly, in the Everglades and Ten Thousand Islands estuaries in south Florida (U.S.), mangrove forests can move landward at the expense of freshwater marshes, but topographic barriers restrict the migration of freshwater marshes into low-lying uplands (Fig. 8 and table S1).

To complicate matters, there is much uncertainty regarding the long-term stability and potential extent of tidal saline wetland migration into adjacent freshwater wetlands. Many of these freshwater wetlands are positioned at elevations that may be equivalent to, below, or just slightly higher than tidal saline wetlands. Thus, these coastal freshwater wetlands are also highly vulnerable to coastal drowning under higher sea level rise rates. Further, in areas with organic-rich biogenic soils, there are critical questions regarding the likelihood of landscape-scale tidal saline migration given the potential for peat collapse and conversion of freshwater wetlands to open water that is too deep for plant establishment and the landward migration of tidal saline wetlands (*39*–*41*). If freshwater wetland soils collapse before tidal saline wetland migration and/or vertical accretion rates do not keep up with sea level rise (*19*–*21*), then the net loss of wetlands could be near the worst-case scenario represented in figs. S1 and S2.

### Where are hot spots for wetland migration into adjacent uplands?

Although most of the landward migration is expected to occur at the expense of freshwater wetlands, there are some estuaries with comparatively large upland areas available for wetland migration (Fig. 8C and table S1). Across the conterminous United States, there are regional differences in the upland land cover classes that are vulnerable to wetland migration (Fig. 6). For example, upland agricultural croplands are especially vulnerable in Louisiana, the mid-Atlantic, central California, and Washington (Fig. 6C). In Louisiana, some of these vulnerable croplands are low-lying rice fields, which are inundated agricultural lands that were formerly seasonal freshwater wetlands and which provide critical avian habitat (*42*) and are often used for seasonal crawfish cultivation.

Upland forests are particularly vulnerable along the low-lying coastal plains of the Atlantic and northern Gulf of Mexico coast (Fig. 6D). In these regions, the conversion of upland forests into ghost forests of dead trees is an especially visible and notable example of the transformative effects of sea level rise (*25*). Upland pasture vulnerability to landward migration is lower than other land cover classes, but there are hot spots where wetlands are expected to migrate into low-lying coastal areas that are managed for domestic livestock (Fig. 6E). For example, low-lying, diked pasturelands in Washington, Oregon, and northern California are particularly vulnerable to landward migration. Coastal Texas has a comparatively high proportion of upland grasslands that are vulnerable to wetland migration (Fig. 6F). Some of these grasslands are coastal prairie ecosystems, which are among the most biologically diverse and endangered plant communities in North America (*43*, *44*). Collectively, these upland-focused analyses explain the regional differences in landscape transformations expected as coastal wetlands migrate into adjacent upland forests, grasslands, agricultural croplands, and pastures.

### Preparing for ecological losses and transformations

Climate change in the form of accelerated sea level rise is already transforming our coastlines, and the pace of those transformations will accelerate in the coming decades (*4*, *5*). Although some tidal saline wetlands can adapt to rising seas via landward migration, our analyses show that those migrations often occur at the expense of valuable freshwater wetlands, agricultural croplands, pastures, terrestrial forests, and grasslands. Thus, there is an urgent need to better anticipate and prepare for the ecological losses and transformations that accompany sea level rise and the landward migration of wetlands. Along the conterminous United States, coastal wetland landward migration will not compensate for the seaward wetland losses that are expected under high rates of sea level rise associated with high-emissions scenarios. Given the value of coastal wetlands globally and the desire to maintain wetland ecosystem services in the face of rising sea levels, similar analyses of the barriers, opportunities, and trade-offs for wetland landward migration are needed across the globe. Understanding and directing the ecological regime shifts and transformative impacts of tidal saline wetland migration into adjacent ecosystems, including highly valued coastal freshwater wetlands and coastal uplands, can help sustain and preserve landscape-scale biodiversity and the ecological and societal benefits provided by coastal ecosystems in the face of rising sea levels.

## MATERIALS AND METHODS

### Study area

We examined the potential for the landward migration of coastal wetlands within the coastal conterminous United States, which includes Washington, DC and 22 coastal states along the Pacific Ocean, Gulf of Mexico, and Atlantic Ocean. Because of regional differences in geomorphology, climate, and management of coastal lands, there is much variation in the structure and function of coastal wetland transitional areas across the United States (Fig. 1). For example, salt marshes and mangrove forests are all tidal saline wetlands that are common in different regions of the United States (*33*). Coastal freshwater wetlands located on the landward side of tidal saline wetlands include tidal and nontidal freshwater wetland forests and marshes (*31*, *37*, *38*). The upland transitional areas adjacent to coastal wetlands include coastal forests, grasslands, agricultural croplands, pastures, and urban lands.

### Global and regional sea level rise

Our analyses examine the effects of a 1.5-m global mean sea level rise scenario, which for 2100, corresponds to the Intermediate-High scenario identified by recent United States interagency sea level rise reports (*5*, *32*). We selected the Intermediate-High scenario to evaluate the potential for wetland migration under a high emissions and high ice sheet loss scenario (*4*, *5*, *32*). For 2150, a 1.5-m global mean sea level rise falls between the Intermediate-Low and Intermediate scenarios (*5*).

Our analyses incorporate data associated with a report that provided global and regional sea level rise scenarios for the Fourth National Climate Assessment (*45*) and the sea level rise chapter (*46*) of the U.S. Global Change Research Program Climate Science Special Report (*47*). In addition to a suite of alternative global mean sea level rise scenarios, the products include regional relative sea level rise scenarios that take into account additional processes that influence local sea level rise rates, such as (i) shifts in oceanographic factors, (ii) changes in gravitational field and rotation, and (iii) vertical land movement (for example, subsidence or uplift) due to sediment compaction, glacial isostatic adjustment, groundwater withdrawals, fossil fuel withdrawals, and other factors (*32*). For our evaluations of potential wetland landward migration under the Intermediate-High scenario (that is, a global mean sea level rise of 1.5 m for 2100), we used the accompanying regional relative sea level rise projections obtained from a 1-degree resolution gridded geospatial dataset (*32*). We then rounded the regional relative sea level rise projections to the nearest 0.1524-m increments, which correspond to the 0.5-ft net sea level increments available for critical wetland migration-related data inputs produced by the National Oceanic and Atmospheric Administration (NOAA) (*48*). At the national scale and for the 1.5-m global mean sea level rise scenario, this process produced regional relative sea level rise projections that ranged from a low of 1.52 m in Washington to a high of 2.59 m in Louisiana.

### Landward migration of coastal wetlands due to sea level rise

Coastal wetlands are resilient ecosystems that have the potential to adapt to sea level rise via two general processes. The first process entails building wetland elevation locally (vertical movement) through biogeomorphic feedbacks between inundation, plant growth, and sedimentation. The second process involves the landward migration (horizontal movement) of wetlands into adjacent upslope or upriver lands. Our analyses focus exclusively on the second process—the landward migration of wetlands.

Our landward migration analyses examine the upslope and upriver movement of two broad wetland classes: (i) tidal saline wetlands and (ii) freshwater wetlands. To our knowledge, there is not a national-scale dataset that quantifies the migration potential of these two broad wetland classes into adjacent freshwater wetland and upland land cover classes. However, as part of the NOAA Sea Level Rise Viewer (https://coast.noaa.gov/slr), the NOAA Office for Coastal Management has produced several related products including national-scale tidal datum data, derived from NOAA’s vertical datum transformation tool (VDatum, https://vdatum.noaa.gov), and Coastal Change Analysis Program (C-CAP) land cover class migration data at 0.1524-m net sea level increments. We obtained these data, which we used in combination with other land cover, elevation, and levee data sources, to identify areas available for the landward migration of tidal saline wetlands and freshwater wetlands.

In our analyses, we made the following assumptions based on definitions provided within NOAA’s Mapping Sea Level Rise Marsh Migration (MSLRMM) effort (*49*): (i) The upper boundary of the future tidal saline wetland zone corresponds to the future position of the Mean High Water Spring (MHWS) tidal datum; (ii) the lower boundary of the future tidal saline wetland zone corresponds to the upper boundary of the current estuarine land cover classes; and (iii) the future freshwater wetland zone occupies the area above the future MHWS tidal datum and below a land cover–based threshold elevation relative to MHWS. This latter threshold, for the upper boundary of future freshwater wetlands, was quantified by NOAA in their MSLRMM analyses.

For most of the country, we used the following: (i) the inland limit of the MSLRMM-derived future estuarine land cover classes (for example, estuarine wetland and brackish/transition wetland) to define the upper boundary of the future tidal saline wetland zone (table S3) and (ii) the MSLRMM-derived future freshwater wetland classes (for example, palustrine forested wetland, palustrine emergent wetland, and palustrine scrub/shrub wetland) to define the upper boundary of the future freshwater wetland zone (table S4). However, for some areas (for example, MSRLMM data gaps or areas with extensive levee or water control systems), we developed complementary analyses using the MSLRMM approach in combination with elevation data and MSLRMM-derived future MHWS data (tables S5 and S6).

To define the lower boundary of the future tidal saline wetland zone, we used the upper boundary of the current estuarine land cover classes (for example, estuarine emergent wetland and estuarine forested wetland; table S7), which was determined using NOAA’s 2016 C-CAP data (*50*). Last, because the landward migration of coastal wetlands is only possible in low-lying lands that are also hydrologically connected to existing coastal wetlands, we applied a 9-pixel connectivity rule to constrain the future wetland migration data. We also used the shrink and expand tool in ArcGIS (Esri, Redlands, CA) to remove small, isolated wetland patches that were low-lying but not connected to coastal wetland migration corridors.

### Estuarine drainage areas

To quantify the potential for landward migration at the estuary level, we developed a geospatial dataset that identifies the boundaries for estuarine drainage areas in the conterminous United States (*51*). Nine estuarine drainage areas in south Florida were delineated using data developed by the South Florida Water Management District (*52*). For the rest of the conterminous United States, we used information contained within the National Fish Habitat Action Plan (NFHAP)–Coastal Spatial Framework (CSF) (*53*). The original NFHAP-CSF data included 612 drainage areas, which were too many for our purposes. Therefore, we merged smaller drainage areas with larger, adjacent drainage areas to reduce the number to 166, which includes 62, 39, and 65 estuarine drainage areas along the Pacific, Gulf of Mexico, and Atlantic coasts, respectively. To ensure that all coastal ecosystems were included, the near-coast estuarine drainage area boundaries were expanded oceanward with a 25-km buffer.

### Data analyses

We quantified the potential area available for tidal saline wetland and freshwater wetland landward migration at multiple spatial scales (i.e., national, coast, state, and estuary). The state and estuary-scale data are available as a U.S. Geological Survey Date Release (*54*). In addition to quantifying the total potential area available for wetland landward migration, we used the 2016 C-CAP data in combination with the future wetland migration data to evaluate the potential for wetland migration into the following six land cover categories: (i) freshwater forest (wetland), (ii) freshwater marsh (wetland), (iii) terrestrial forest (upland), (iv) terrestrial grassland (upland), (v) agricultural croplands (upland), and (vi) pasture (upland). We also consolidated these categories to quantify potential migration into upland and wetland classes. We use percentages of total wetland migration to convey the relative vulnerability of different land cover classes to wetland migration. Last, we used the current C-CAP cover classes to quantify the current tidal saline wetland area and the current freshwater wetland area that is vulnerable to future tidal saline wetland migration. We used these data at the estuary level to characterize the relationships between (i) the current tidal saline wetland area to the total area available for future tidal saline wetland migration (i.e., uplands and freshwater wetlands), (ii) the current tidal saline wetland area to the area available for future tidal saline wetland migration into freshwater wetlands, and (iii) the current freshwater wetland area vulnerable to future tidal saline wetland migration compared to the area available for future freshwater migration into uplands.
