# Supplementary material for: Migration and transformation of coastal wetlands in response to rising seas
Source: Sci Adv. 2022 Jun 29;8(26):eabo5174. doi: 10.1126/sciadv.abo5174 (PMC9242587; doi:10.1126/sciadv.abo5174)
Supplement: Supplementary file 1 — Figs. S1 and S2 Tables S1 to S7 [file sciadv.abo5174_sm.pdf]

Science Advances
AAAS

# Supplementary Materials for

## Migration and transformation of coastal wetlands in response to rising seas

Michael J. Osland *et al.*

Corresponding author: Michael J. Osland, mosland@usgs.gov

*Sci. Adv.* **8**, eabo5174 (2022)
DOI: 10.1126/sciadv.abo5174

**This PDF file includes:**

Figs. S1 and S2
Tables S1 to S7

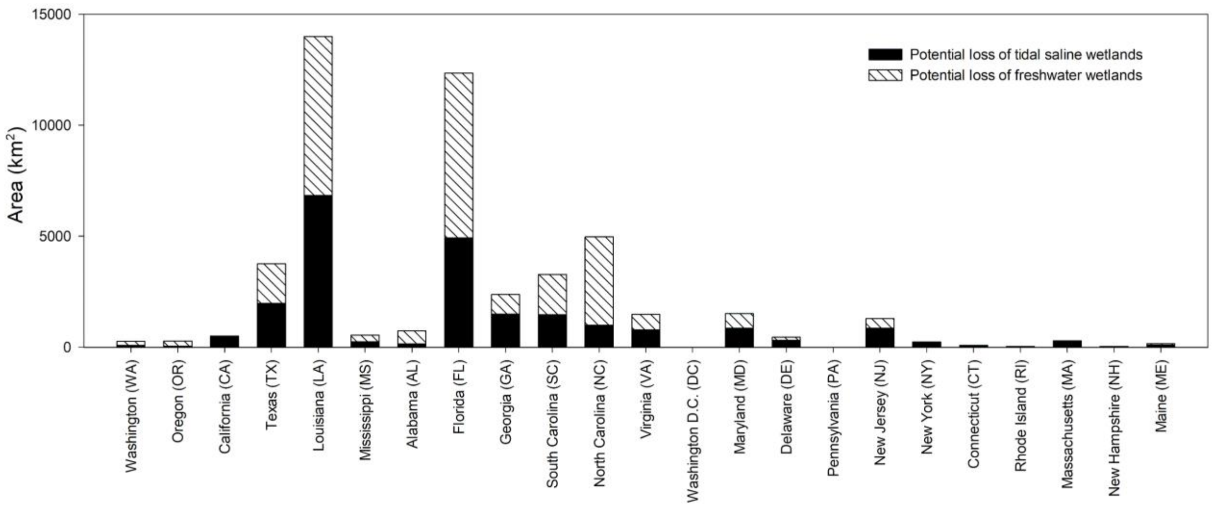


**Fig. S1. The potential loss of coastal wetlands due to rising sea levels across the conterminous United States, within 22 coastal states and Washington D.C.** These losses are for a worst-case scenario where biogeomorphic feedbacks are not able to compensate for high rates of sea-level rise as thresholds for vertical accretion are surpassed (*19-21*) and saltwater intrusion leads to the collapse of freshwater wetlands (*39-41*).

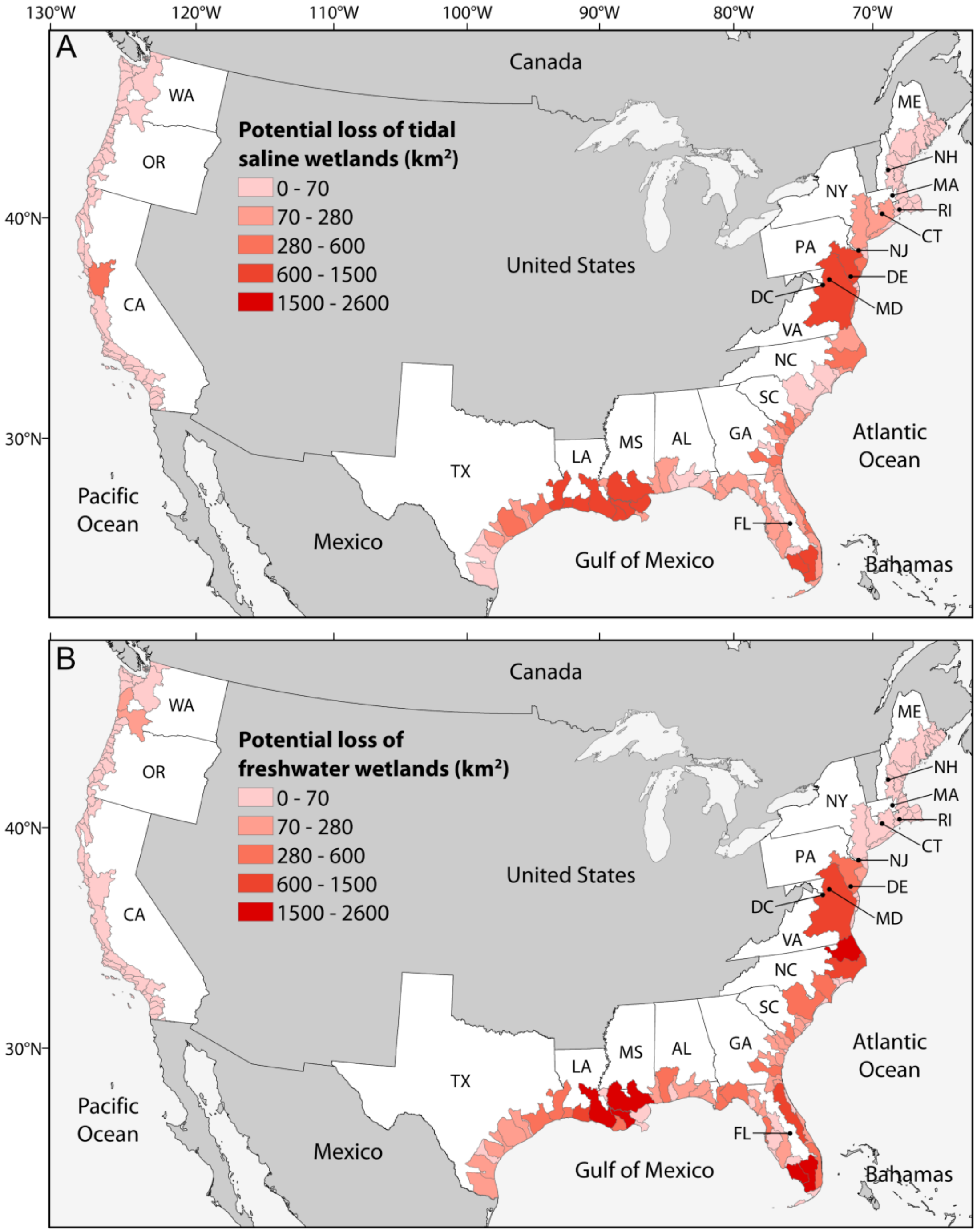


**Fig. S2. Maps of potential loss of tidal saline and freshwater wetlands across the conterminous United States, within 166 estuarine drainage areas.** (**A**) Map of potential areal loss of tidal saline wetlands within estuarine drainage areas. (**B**) Map of potential areal loss of freshwater wetlands within estuarine drainage areas. These losses are for a worst-case scenario where biogeomorphic feedbacks are not able to compensate for high rates of sea-level rise as thresholds for vertical accretion are surpassed (*19-21*) and saltwater intrusion leads to the collapse of freshwater wetlands (*39-41*). Note that the 166 polygons on the maps are estuarine drainage areas and the colors reflect the amount of potential loss within each polygon but not the inland extent of potential loss.

**Table S1. Estuarine drainage area with the largest area available for coastal wetland migration across the conterminous United States.** The top 30 estuarine drainage areas are shown in descending ranked order.

| Estuary (State[s]) | Area available for coastal wetland migration ($km^2$; rank) | Area available for tidal saline wetland migration into freshwater wetlands ($km^2$; rank) | Area available for tidal saline wetland migration into uplands ($km^2$; rank) | Area available for freshwater wetland migration into uplands ($km^2$; rank) |
|---|---|---|---|---|
| Albemarle (NC, VA) | 3815 (1) | 2537 (1) | 1082 (3) | 197 (1) |
| Mermentau (LA) | 2876 (2) | 1422 (7) | 1311 (1) | 143 (3) |
| Atchafalaya-Vermilion (LA) | 2698 (3) | 1732 (4) | 866 (5) | 100 (6) |
| Pamlico (NC) | 2465 (4) | 1210 (8) | 1094 (2) | 161 (2) |
| Chesapeake (VA, MD) | 2127 (5) | 1088 (9) | 896 (4) | 142 (4) |
| Barataria (LA) | 2040 (6) | 1685 (5) | 326 (9) | 28 (21) |
| West Mississippi Sound (LA, MS) | 2037 (7) | 1678 (6) | 299 (10) | 61 (13) |
| Big Cypress (FL) | 1960 (8) | 1828 (2) | 104 (26) | 28 (22) |
| Everglades (FL) | 1870 (9) | 1809 (3) | 61 (39) | 1 (93) |
| St. Johns (FL) | 1122 (10) | 838 (10) | 212 (14) | 72 (9) |
| Sabine (TX, LA) | 921 (11) | 516 (13) | 340 (7) | 64 (12) |
| Galveston (TX) | 892 (12) | 483 (15) | 335 (8) | 74 (8) |
| Calcasieu Lake (LA) | 722 (13) | 285 (24) | 369 (6) | 68 (11) |
| Delaware (DE, NJ, PA) | 709 (14) | 364 (19) | 299 (11) | 46 (15) |
| St. Helena (SC) | 679 (15) | 437 (16) | 205 (15) | 36 (17) |
| Southeast Florida (FL) | 605 (16) | 340 (20) | 134 (19) | 131 (5) |
| Winyah (SC) | 575 (17) | 494 (14) | 65 (38) | 16 (29) |
| Apalachicola (FL) | 574 (18) | 517 (12) | 48 (43) | 9 (42) |

| | | | | |
|---|---|---|---|---|
| Mobile (AL) | 558 (19) | 528 (11) | 24 (62) | 6 (50) |
| Apalachee (FL) | 544 (20) | 405 (17) | 105 (25) | 34 (19) |
| St. Andrew-St. Simons (GA) | 527 (21) | 289 (23) | 179 (17) | 59 (14) |
| Terrebonne-Timbalier (LA) | 503 (23) | 370 (18) | 131 (21) | 1 (82) |
| Matagorda (TX) | 502 (22) | 175 (33) | 234 (12) | 93 (7) |
| Upper Laguna Madre (TX) | 404 (24) | 108 (42) | 228 (13) | 68 (10) |
| St. Catherines-Sapelo (GA) | 392 (25) | 223 (27) | 132 (20) | 36 (18) |
| Stono-North Edisto (SC) | 386 (26) | 171 (34) | 182 (16) | 32 (20) |
| Crystal-Pithlachascotee (FL) | 357 (27) | 290 (22) | 52 (41) | 14 (32) |
| Suwannee (FL) | 353 (28) | 259 (25) | 77 (35) | 16 (30) |
| Lower Laguna Madre (TX) | 347 (29) | 209 (28) | 95 (29) | 43 (16) |
| Cape Fear (NC) | 341 (30) | 292 (21) | 39 (50) | 10 (39) |

**Table S2. Estuarine drainage areas with the largest area available for coastal wetland migration along the Pacific coast of the conterminous United States.** The top ten estuarine drainage areas along the Pacific coast are shown in descending ranked order.

| Estuary (State[s]) | Area available for coastal wetland migration ($km^2$; rank) | Area available for tidal saline wetland migration into freshwater wetlands ($km^2$; rank) | Area available for tidal saline wetland migration into uplands ($km^2$; rank) | Area available for freshwater wetland migration into uplands ($km^2$; rank) |
|---|---|---|---|---|
| Columbia (OR, WA) | 233 (36) | 143 (36) | 83 (33) | 7 (46) |
| Puget (WA) | 190 (42) | 34 (63) | 147 (18) | 10 (40) |
| Willapa-Grays (WA) | 134 (53) | 83 (49) | 45 (45) | 7 (47) |
| Coquille (OR) | 45 (67) | 23 (68) | 21 (67) | 1 (87) |
| Eel (CA) | 43 (68) | 5 (100) | 35 (51) | 3 (66) |
| Coos (OR) | 38 (70) | 23 (70) | 13 (79) | 2 (77) |
| Elkhorn (CA) | 37 (72) | 6 (93) | 25 (60) | 5 (54) |
| Humboldt (CA) | 31 (77) | 4 (105) | 25 (61) | 2 (80) |
| Nooksack (WA) | 30 (80) | 8 (84) | 21 (68) | 1 (89) |
| San Francisco (CA) | 27 (83) | 15 (77) | 9 (85) | 2 (76) |

**Table S3. Land cover classes used to define the upper boundary of the future tidal saline wetland zone.** These land cover classes are from the National Oceanic and Atmospheric Administration (NOAA) Mapping Sea Level Rise Marsh Migration (MSLRMM) effort (*49*). Landward migration of coastal wetlands is only possible in low-lying lands that are also hydrologically connected to existing coastal wetlands. Therefore, we applied a connectivity rule to these future land cover classes.

| **Land cover class name** | **Source** |
|---|---|
| Brackish/Transition Wetland | NOAA MSLRMM |
| Estuarine Wetland | NOAA MSLRMM |
| Unconsolidated Shore | NOAA MSLRMM |
| Open Water | NOAA MSLRMM |

**Table S4. Land cover classes used to define the upper boundary of the future freshwater wetland zone.** These land cover classes are from the National Oceanic and Atmospheric Administration (NOAA) Mapping Sea Level Rise Marsh Migration (MSLRMM) effort (*49*). Landward migration of coastal wetlands is only possible in low-lying lands that are also hydrologically connected to existing coastal wetlands. Therefore, we applied a connectivity rule to these future land cover classes.

| **Land cover class name** | **Source** |
|---|---|
| Palustrine Forested Wetland | NOAA MSLRMM |
| Palustrine Scrub/Shrub Wetland | NOAA MSLRMM |
| Palustrine Emergent Wetland | NOAA MSLRMM |

**Table S5. Areas with gaps in National Oceanic and Atmospheric Administration (NOAA) Mapping Sea Level Rise Marsh Migration (MSLRMM) data (*49*).** We developed complementary analyses for these areas using the MSLRMM approach in combination with elevation data and MSLRMM-derived future Mean High Water Spring (MHWS) tidal datum data.

| **Location** |
| --- |
| Northwest Washington |
| Central-west Florida |
| South Florida |
| Northeast Florida |

**Table S6. Areas where complementary analyses were developed due to the presence of extensive levee or water control systems that were not incorporated in the original National Oceanic and Atmospheric Administration (NOAA) Mapping Sea Level Rise Marsh Migration (MSLRMM) analyses (*49*).** For these areas, we: (1) developed complementary analyses using the MSLRMM approach in combination with elevation data and MSLRMM-derived future Mean High Water Spring (MHWS) tidal datum data; and/or (2) used levee or water control structure boundaries to restrict wetland migration.

| Estuarine Drainage Area(s) | State(s) |
|---|---|
| Puget Sound and Nooksack | Washington |
| Columbia River | Oregon and Washington |
| Humboldt Bay and Eel River | California |
| San Francisco | California |
| All | Louisiana |
| Everglades and Big Cypress Swamp | Florida |
| Delaware Bay | Delaware, New Jersey, Pennsylvania |

**Table S7. To define the lower boundary of the future tidal saline wetland zone, we used the upper boundary of the following current estuarine land cover classes determined using the National Oceanic and Atmospheric Administration (NOAA) 2016 Coastal Change Analysis Program (C-CAP) land cover data (*50*).**

| **Land cover class name** |
|---|
| Estuarine Forested Wetland |
| Estuarine Scrub/Shrub Wetland |
| Estuarine Emergent Wetland |
| Estuarine Aquatic Bed |
| Unconsolidated Shore |
| Open Water |
